# Preparedness, Satisfaction, and Intentions Among Dental Students Treating Patients With Disabilities: Evidence From Two Mexican Dental Schools

**DOI:** 10.7759/cureus.86737

**Published:** 2025-06-25

**Authors:** Pedro Rosales García, Grecia Carolina Medina Morales, Alan Martínez Zumarán, Adriana Torre Delgadillo, Carlos Bermúdez Jiménez, Joshua Hernández Benavidez, Miguel Angel Casillas Santana, Oscar Lozano González, Nuria Patiño-Marín, Marco Felipe Salas Orozco

**Affiliations:** 1 Faculty of Stomatology of the Northeastern Regional Complex, Meritorious Autonomous University of Puebla, Puebla, MEX; 2 Dentistry, Clinical Research Laboratory, Faculty of Stomatology, Autonomous University of San Luis Potosí, San Luis Potosí, MEX; 3 Specialty in Orthodontics and Dentomaxillofacial Orthopedics, Faculty of Stomatology, Autonomous University of San Luis Potosí, San Luis Potosí, MEX; 4 Academic Unit of Dentistry, Autonomous University of Zacatecas, Zacatecas, MEX; 5 Stomatology with Terminal Option in Orthodontics, Faculty of Stomatology, Meritorious Autonomous University of Puebla, Puebla, MEX; 6 Clinical Research Laboratory, Faculty of Stomatology, Autonomous University of San Luis Potosí, San Luis Potosí, MEX; 7 Dentistry, Autonomous University of San Luis Potosí, San Luis Potosí, MEX

**Keywords:** attitudes and intentions, dental education, inclusive dental care, mexican dental schools, special care dentistry

## Abstract

Background: The inclusion of people with disabilities in oral healthcare is increasingly recognized as a global priority. Despite this, individuals with disabilities continue to face significant barriers to dental care, often due to a lack of specialized training among providers. Dental education plays a crucial role in preparing future practitioners to deliver inclusive and equitable care.

Objective: This study aimed to assess the perceptions, satisfaction, and professional intentions of dental students regarding their preparedness to treat patients with disabilities, and to compare these perceptions between two Mexican dental schools.

Methods: A cross-sectional survey was conducted among undergraduate dental students from the third to sixth year at two institutions: the Faculty of Stomatology at the Universidad Autónoma de San Luis Potosí (UASLP) and the Faculty of Dentistry at the Northeastern Regional Complex of the Meritorious Autonomous University of Puebla (BUAP). A validated 14-item questionnaire was adapted and administered in person, reaching over 90% of the eligible population. Statistical analysis was performed using JASP software, applying chi-square tests to identify institutional differences in attitudes, perceptions, satisfaction, and behavioral intentions.

Results: A total of 502 students participated (248 (49.4%) from UASLP and 254 (50.6%) from BUAP). Female students comprised 201 (81.0%) at UASLP and 181 (71.3%) at BUAP, while male students represented 47 (19.0%) at UASLP and 73 (28.7%) at BUAP. BUAP students consistently reported significantly higher satisfaction with classroom, clinical, and extramural experiences, and greater confidence in treating patients with disabilities (p < 0.001). Differences were also found in perceived institutional support and faculty expertise. While both groups acknowledged the importance of improving disability-related education, 207 (81.5%) BUAP students expressed stronger behavioral intentions to include patients with disabilities in their future practice compared to 156 (62.9%) from UASLP (p = 0.00095).

Conclusion: Despite a shared recognition of the importance of disability care, significant institutional differences were found in students' preparedness and educational experiences. These findings highlight the urgent need to enhance dental curricula with structured, experiential training and faculty development to ensure that future professionals are equipped to provide ethical and inclusive care to all patient populations.

## Introduction

Individuals with special health care needs (SHCN) - defined as those who have or are at increased risk for a chronic physical, developmental, behavioral, or emotional condition and who also require health and related services beyond that required by children or adults generally - often encounter significant challenges in accessing equitable healthcare, including oral health services. The inclusion of people with disabilities in all areas of healthcare has become an increasingly prominent global concern, with particular emphasis on the need for accessible and equitable oral health services. Individuals with disabilities often face numerous barriers to receiving adequate dental care, including physical limitations, transportation challenges, stigma, and a lack of trained professionals [1]. In dentistry, these barriers are further compounded by limited curricular exposure and inadequate training among students and practitioners in managing the needs of individuals with physical, intellectual, sensory, or multiple disabilities [2].

Disability, defined by the World Health Organization (WHO) as a dynamic interaction between health conditions and contextual factors, affects more than one billion people globally [3]. In Mexico, national surveys estimate that approximately 6 to 8 million individuals live with a disability, representing a substantial segment of the population requiring specialized healthcare services [4]. Despite increasing recognition of this need, dental care remains one of the most unmet health needs among individuals with disabilities - particularly in regional and urban centers where health disparities persist and access to specialized dental services is limited [5].

The successful treatment of patients with disabilities requires not only technical competence but also appropriate attitudes, effective communication skills, and strong ethical awareness. Future dental professionals must be equipped to recognize the importance of inclusive care and to develop the confidence and practical abilities necessary to manage a diverse range of patient needs. Accordingly, dental education programs should integrate both theoretical and practical components of special care dentistry, emphasizing experiential learning, clinical exposure, and interprofessional collaboration [6].

In recent years, numerous studies worldwide have reported that dental students often feel unprepared to treat patients with disabilities. The most commonly cited barriers include a lack of hands-on experience, the absence of dedicated training modules, and insufficient faculty support. These factors contribute to a sense of insecurity among students, which may ultimately affect their willingness and ability to treat such patients in future clinical practice. This challenge is particularly evident in regions where dental curricula have not yet fully integrated disability-focused training or where opportunities for interaction with patients with special needs are limited during undergraduate education [7,8].

This study aims to assess the perceptions of dental students from two Mexican dental schools regarding their readiness to treat patients with disabilities. By examining students’ views on the quality and adequacy of their training, the extent of their clinical exposure to patients with disabilities, and their confidence in providing care, the study seeks to inform educators and policymakers about current educational practices and potential areas for improvement.

## Materials and methods

This cross-sectional, observational study was conducted in the city of San Luis Potosí, Mexico, within an undergraduate dental surgery program. The study protocol was reviewed and approved by the Research Ethics Committee of the Faculty of Stomatology at the Universidad Autónoma de San Luis Potosí (UASLP) under reference number CEI-FE-008-024. The protocol was evaluated for ethical, legal, and biosafety compliance and received unanimous approval from the Committee. The Committee operates in accordance with national guidelines established by the National Bioethics Commission (CONBIOÉTICA) and is registered under code CONBIOÉTICA-24-CEI-001-20190213. All procedures adhered to the ethical standards set forth in the Declaration of Helsinki.

The survey used in this study was adapted from a previously published instrument developed based on validated questionnaires from earlier research [9-12]. The original draft was designed by the investigators and included additional items addressing clinical services for patients with special needs. It was pilot tested with students, staff, and faculty members of the Multicultural Affairs Committee at the University of Michigan School of Dentistry. Feedback from this pilot was incorporated into the final version to enhance content validity and ensure alignment with the study objectives [13]. Permission was formally obtained from John Wiley and Sons to reuse specific portions of previously published questionnaires contained in five articles referenced in this study [9-13]. These include extracts from tables and instruments originally published in the Journal of Dental Education, as follows: (1) “Dental Education and Care for Underserved Patients: An Analysis of Students’ Intentions and Alumni Behavior” (License No. 6042311277617), (2) “General Dentists and Pediatric Dental Patients: The Role of Dental Education” (License No. 6042320257012), (3) “Orthodontists’ and Orthodontic Residents’ Education in Treating Underserved Patients” (License No. 6042320683759), (4) “General Dentists and Special Needs Patients: Does Dental Education Matter?” (License No. 6042320948529), and (5) “Patients with Special Needs: Dental Students’ Educational Experiences, Attitudes, and Behavior” (License No. 6044491492609). All reused content has been appropriately cited and is included in the appendices of this manuscript. The reuse was authorized for non-commercial academic purposes in accordance with Wiley’s licensing terms and the Copyright Clearance Center.

The final version of the questionnaire, comprising 18 items (Appendices: Table 7), was administered in person during class sessions to dental students in their third through sixth years at the Faculty of Stomatology, UASLP, and the Faculty of Dentistry, Northeastern Regional Complex, Benemérita Universidad Autónoma de Puebla (BUAP). The instrument reached over 90% of the student population enrolled at those academic levels in each institution. This high response rate ensured the robustness of the dataset and enhanced the representativeness and generalizability of the findings. Permission was first obtained from the course instructors to administer the survey during class hours. Researchers then visited the classrooms in person and provided students with a link to an online questionnaire hosted on the Google Forms platform. The first page of the form presented the informed consent statement, and students could proceed only if they agreed to participate voluntarily. This procedure ensured ethical compliance and preserved participant autonomy.

The questionnaire was structured into three main sections. The first section collected demographic and academic background information, ensuring anonymity by omitting identifying questions, such as names. The second section explored students’ perceptions regarding the quality of their education and their satisfaction with the training they received in caring for patients with disabilities. These questions addressed students’ perceived competence, exposure to relevant clinical experiences, and self-assessed readiness to manage such cases in professional settings. The third section focused on future intentions, specifically investigating whether students intended to treat patients with disabilities in their future private practices. Although the instrument was adapted from previously published sources, formal permission to use the instrument was not sought, as the study was conducted solely for academic and non-commercial purposes. Proper attribution was maintained in accordance with accepted scholarly standards for reusing research instruments.

Eligibility for participation was determined using specific inclusion, exclusion, and elimination criteria. Inclusion criteria comprised students currently enrolled in the dental surgery program, regardless of gender, from the third through the sixth year of study. All participants were required to have prior clinical experience involving direct patient care and to provide informed consent for voluntary participation. Students in the first and second academic years, as well as those who had not yet engaged in clinical practice, were excluded. Additionally, students who were not enrolled at the time of the study or who declined to provide consent were excluded. Lastly, incomplete questionnaires or those lacking sufficient data for analysis were eliminated from the dataset.

Statistical analysis

The statistical analysis was conducted using JASP software. Descriptive statistics were calculated to summarize the frequency and percentage distributions of students' responses across the different institutions (UASLP and BUAP) and academic years. To evaluate differences in students’ perceptions, attitudes, satisfaction levels, and behavioral intentions between the two institutions, chi-square tests of independence (χ²) were applied to each of the categorical variables. A p-value of < 0.05 was considered statistically significant.

## Results

Table 1 summarizes the demographic characteristics of the dental students who participated in the study from the UASLP and the BUAP. The table presents the distribution by sex, average academic year by sex, age by academic year, and overall age range.

**Table 1:** Demographic characteristics of dental students from UASLP and BUAP — indicates that no information is applicable or required for that specific cell. It is used to explicitly mark the absence of data where a value or subcategory does not apply. Values are expressed as frequency and percentage: N (%). UASLP: Universidad Autónoma de San Luis Potosí; BUAP: Benemérita Universidad Autónoma de Puebla

Category	Subcategory	UASLP (n = 248)	BUAP (n = 254)
1. Sex	Female	201 (81.0%)	181 (71.3%)
	Male	47 (19.0%)	73 (28.7%)
2. Academic year (Mean, SD)	Female	2.64 (1.12)	2.41 (1.20)
	Male	2.68 (1.07)	2.40 (1.20)
3. Age by academic year	3rd year	21.18 (1.37), n = 51	20.96 (1.25), n = 71
	4th year	21.79 (1.48), n = 58	21.89 (1.60), n = 88
	5th year	23.06 (1.53), n = 66	23.87 (1.64), n = 15
	6th year	23.78 (1.13), n = 73	24.23 (2.36), n = 80
4. Age range (years)	—	18–28	19–33

The results from Table 2 indicate that students from BUAP consistently reported more positive perceptions across all three items when compared to their counterparts at UASLP, highlighting potential institutional differences in training approaches, clinical environments, and emphasis on the care of patients with special needs.

**Table 2:** Perceptions about dental education and the clinical environment “N” represents the number of respondents in each academic year. Percentages (%) are calculated within each academic year group. Values are expressed as frequency and percentage: N (%). UASLP: Universidad Autónoma de San Luis Potosí; BUAP: Benemérita Universidad Autónoma de Puebla

Category	Subcategory	UASLP – 3rd year (N = 51)	UASLP – 4th year (N = 58)	UASLP – 5th year (N = 66)	UASLP – 6th year (N = 73)	BUAP – 3rd year (N = 71)	BUAP – 4th year (N = 88)	BUAP – 5th year (N = 15)	BUAP – 6th year (N = 80)	p-value
5. I believe my dental school has a genuine interest/concern for treating patients with special needs	Strongly disagree	1 (2.0%)	2 (3.5%)	7 (10.6%)	9 (9.6%)	0 (0.0%)	1 (1.1%)	0 (0.0%)	8 (10.0%)	<0.001
	Disagree	14 (27.5%)	19 (32.8%)	27 (40.9%)	35 (12.3%)	5 (7.0%)	13 (14.8%)	4 (26.7%)	7 (8.8%)	
	Neutral	19 (37.3%)	27 (46.6%)	19 (28.8%)	12 (48.0%)	22 (31.0%)	32 (36.4%)	6 (40.0%)	27 (33.8%)	
	Agree	14 (27.5%)	10 (17.2%)	11 (16.7%)	14 (16.4%)	37 (52.1%)	33 (37.5%)	3 (20.0%)	24 (30.0%)	
	Strongly agree	3 (5.9%)	0 (0.0%)	2 (3.0%)	3 (19.2%)	7 (9.9%)	9 (10.2%)	2 (13.3%)	14 (17.5%)	
6. My classes prepared me well to treat patients with special needs	Strongly disagree	1 (2.0%)	10 (17.2%)	10 (15.2%)	7 (9.6%)	0 (0.0%)	1 (1.1%)	1 (6.7%)	3 (3.8%)	<0.001
	Disagree	19 (37.3%)	19 (32.8%)	28 (42.4%)	36 (49.3%)	2 (2.8%)	19 (21.6%)	2 (13.3%)	6 (7.5%)	
	Neutral	20 (39.2%)	20 (34.5%)	18 (27.3%)	10 (13.7%)	47 (66.2%)	45 (51.1%)	7 (46.7%)	36 (45.0%)	
	Agree	10 (19.6%)	9 (15.5%)	5 (7.6%)	12 (16.4%)	19 (26.8%)	16 (18.2%)	5 (33.3%)	25 (31.3%)	
	Strongly agree	1 (2.0%)	0 (0.0%)	5 (7.6%)	8 (11.0%)	3 (4.2%)	7 (8.0%)	0 (0.0%)	10 (12.5%)	
7. The school’s clinics provide a sensitive environment for treating patients with special needs	Strongly disagree	4 (7.8%)	7 (12.1%)	10 (10.6%)	11 (15.1%)	0 (0.0%)	4 (4.6%)	0 (0.0%)	7 (8.8%)	<0.001
	Disagree	14 (27.5%)	18 (31.0%)	29 (15.2%)	29 (39.7%)	7 (9.9%)	8 (9.1%)	5 (33.3%)	9 (11.3%)	
	Neutral	19 (37.3%)	25 (43.1%)	17 (43.9%)	19 (26.0%)	26 (36.6%)	43 (48.9%)	5 (33.3%)	25 (31.3%)	
	Agree	14 (27.5%)	7 (12.1%)	7 (10.6%)	11 (15.1%)	32 (45.1%)	30 (34.1%)	3 (20.0%)	28 (35.0%)	
	Strongly agree	0 (0.0%)	1 (1.7%)	3 (4.5%)	3 (4.1%)	6 (8.5%)	3 (3.4%)	2 (13.3%)	11 (13.8%)	

Table 3 shows significant differences between UASLP and BUAP students in their educational attitudes toward treating patients with special needs. Statistically significant differences were found in the perception that the curriculum should include more education (p < 0.001) and in the importance placed on educating students about this topic (p = 0.0007). However, no significant difference was observed regarding the desire to have one more year of residency before feeling comfortable treating such patients (p = 0.067), indicating similar levels of perceived preparedness between both institutions.

**Table 3:** Education-related attitudes “N” indicates the number of respondents per academic year. Percentages (%) represent the proportion of students within each academic year group. Values are expressed as frequency and percentage: N (%). UASLP: Universidad Autónoma de San Luis Potosí; BUAP: Benemérita Universidad Autónoma de Puebla

Category	Subcategory	UASLP – 3rd year (N = 51)	UASLP – 4th year (N = 58)	UASLP – 5th year (N = 66)	UASLP – 6th year (N = 73)	BUAP – 3rd year (N = 71)	BUAP – 4th year (N = 88)	BUAP – 5th year (N = 15)	BUAP – 6th year (N = 80)	p-value
8. The curriculum should include more education about treating patients with special needs	Strongly disagree	0 (0.0%)	2 (3.5%)	2 (3.0%)	2 (2.7%)	0 (0.0%)	0 (0.0%)	0 (0.0%)	4 (5.0%)	<0.01
	Disagree	0 (0.0%)	1 (1.7%)	2 (3.0%)	0 (0.0%)	2 (2.8%)	1 (1.1%)	0 (0.0%)	0 (0.0%)	
	Neutral	4 (7.8%)	3 (5.2%)	8 (12.1%)	6 (8.2%)	12 (16.9%)	13 (14.8%)	2 (13.3%)	7 (8.8%)	
	Agree	20 (39.2%)	23 (39.7%)	9 (13.6%)	19 (26.0%)	42 (59.2%)	42 (47.7%)	5 (33.3%)	31 (38.8%)	
	Strongly agree	27 (52.9%)	29 (50.0%)	45 (68.2%)	46 (63.0%)	15 (21.1%)	32 (36.4%)	8 (53.3%)	38 (47.5%)	
9. It is very important to educate students about the treatment of patients with special needs	Strongly disagree	0 (0.0%)	2 (3.5%)	2 (3.0%)	4 (5.5%)	0 (0.0%)	0 (0.0%)	0 (0.0%)	4 (5.0%)	<0.01
	Disagree	0 (0.0%)	0 (0.0%)	1 (1.5%)	3 (4.1%)	0 (0.0%)	0 (0.0%)	0 (0.0%)	0 (0.0%)	
	Neutral	2 (3.9%)	3 (5.2%)	4 (6.1%)	3 (4.1%)	1 (1.4%)	8 (9.1%)	1 (6.7%)	3 (3.8%)	
	Agree	17 (33.3%)	15 (25.9%)	13 (19.7%)	14 (19.2%)	34 (47.9%)	32 (36.4%)	3 (20.0%)	28 (35.0%)	
	Strongly agree	32 (62.8%)	38 (65.5%)	46 (69.7%)	49 (67.1%)	36 (50.7%)	48 (54.5%)	11 (73.3%)	45 (56.3%)	
10. I would like to have one more year as a resident before I feel comfortable treating patients with special needs	Strongly disagree	4 (7.8%)	5 (8.6%)	5 (7.6%)	9 (12.3%)	1 (1.4%)	8 (9.1%)	0 (0.0%)	5 (6.3%)	0.067
	Disagree	5 (9.8%)	6 (10.3%)	8 (12.1%)	7 (9.6%)	4 (5.6%)	11 (12.5%)	2 (13.3%)	5 (6.3%)	
	Neutral	22 (43.1%)	19 (32.8%)	28 (42.4%)	26 (35.6%)	28 (39.4%)	31 (35.2%)	6 (40.0%)	28 (35.0%)	
	Agree	18 (35.3%)	19 (32.8%)	16 (24.2%)	24 (32.9%)	33 (46.5%)	24 (27.3%)	3 (20.0%)	27 (33.8%)	
	Strongly agree	2 (3.9%)	9 (15.5%)	9 (13.6%)	7 (9.6%)	5 (7.0%)	14 (15.9%)	4 (26.7%)	15 (18.8%)	

The chi-square analysis of Table 4 revealed statistically significant differences between UASLP and BUAP students in their satisfaction with classroom experience (p = 0.0003), clinical experience (p < 0.0001), extramural experience (p < 0.0001), and faculty expertise (p = 0.0002). However, no significant difference was observed in the first assessment of satisfaction with the patient pool (p = 0.086), indicating comparable levels of perceived access to diverse patient cases between both institutions.

**Table 4:** Satisfaction with various aspects of education about treating patients with special health care needs “N” represents the number of respondents per academic year. Percentages (%) were calculated within each academic year group. Values are expressed as frequency and percentage: N (%). UASLP: Universidad Autónoma de San Luis Potosí; BUAP: Benemérita Universidad Autónoma de Puebla

Category	Subcategory	UASLP – 3rd year (N = 51)	UASLP – 4th year (N = 58)	UASLP – 5th year (N = 66)	UASLP – 6th year (N = 73)	BUAP – 3rd year (N = 71)	BUAP – 4th year (N = 88)	BUAP – 5th year (N = 15)	BUAP – 6th year (N = 80)	p-value
11. Satisfaction with classroom experience	Very Dissatisfied	2 (3.9%)	2 (3.4%)	8 (12.1%)	7 (2.7%)	0 (0.0%)	2 (2.3%)	0 (0.0%)	4 (5.0%)	<0.01
	Dissatisfied	7 (13.7%)	13 (22.4%)	20 (30.3%)	2 (21.9%)	0 (0.0%)	15 (17.1%)	3 (20.0%)	5 (6.3%)	
	Neutral	31 (60.8%)	35 (60.3%)	25 (37.9%)	16 (46.6%)	37 (52.1%)	46 (52.3%)	7 (46.7%)	41 (51.3%)	
	Satisfied	10 (19.6%)	8 (13.8%)	12 (18.2%)	34 (23.3%)	34 (47.9%)	24 (27.3%)	4 (26.7%)	23 (28.8%)	
	Very Satisfied	1 (2.0%)	0 (0.0%)	1 (1.5%)	17 (5.5%)	0 (0.0%)	1 (1.1%)	1 (6.7%)	7 (8.8%)	
12. Satisfaction with clinical experience	Very Dissatisfied	2 (3.9%)	0 (0.0%)	3 (4.6%)	2 (2.7%)	0 (0.0%)	3 (3.4%)	0 (0.0%)	3 (3.8%)	<0.01
	Dissatisfied	9 (17.6%)	16 (27.6%)	23 (34.8%)	33 (45.2%)	4 (5.6%)	10 (11.4%)	3 (20.0%)	9 (11.3%)	
	Neutral	23 (45.1%)	29 (50.0%)	24 (36.4%)	20 (27.4%)	47 (66.2%)	52 (59.1%)	8 (53.3%)	33 (41.3%)	
	Satisfied	14 (27.5%)	10 (17.2%)	7 (10.6%)	9 (12.3%)	19 (26.8%)	20 (22.7%)	3 (20.0%)	27 (33.8%)	
	Very Satisfied	3 (5.9%)	3 (5.2%)	9 (13.6%)	9 (12.3%)	1 (1.4%)	3 (3.4%)	1 (6.7%)	8 (10.0%)	
13. Satisfaction with extramural experience	Very Dissatisfied	7 (13.7%)	4 (6.9%)	9 (13.6%)	23 (31.5%)	0 (0.0%)	3 (3.4%)	1 (6.7%)	3 (3.8%)	<0.01
	Dissatisfied	6 (11.8%)	16 (27.6%)	20 (30.3%)	20 (27.4%)	4 (5.6%)	6 (6.8%)	1 (6.7%)	5 (6.3%)	
	Neutral	25 (49.0%)	26 (44.8%)	26 (39.4%)	20 (27.4%)	48 (67.6%)	61 (69.3%)	9 (60.0%)	32 (40.0%)	
	Satisfied	12 (23.5%)	11 (19.0%)	10 (15.2%)	8 (11.0%)	18 (25.4%)	17 (19.3%)	3 (20.0%)	34 (42.5%)	
	Very Satisfied	1 (2.0%)	1 (1.7%)	1 (1.5%)	2 (2.7%)	1 (1.4%)	1 (1.1%)	1 (6.7%)	6 (7.5%)	
14. Satisfaction with faculty expertise	Very Dissatisfied	0 (0.0%)	0 (0.0%)	5 (7.6%)	4 (5.5%)	0 (0.0%)	2 (2.3%)	1 (6.7%)	2 (2.5%)	<0.01
	Dissatisfied	4 (7.8%)	16 (27.6%)	21 (31.8%)	11 (15.1%)	5 (7.0%)	6 (6.8%)	3 (20.0%)	7 (8.8%)	
	Neutral	19 (37.3%)	27 (46.6%)	21 (31.8%)	27 (37.0%)	41 (57.8%)	52 (59.1%)	8 (53.3%)	30 (37.5%)	
	Satisfied	25 (49.0%)	15 (25.9%)	18 (27.3%)	27 (37.0%)	23 (32.4%)	24 (27.3%)	2 (13.3%)	33 (41.3%)	
	Very Satisfied	3 (5.9%)	0 (0.0%)	1 (1.5%)	4 (5.5%)	2 (2.8%)	4 (4.6%)	1 (6.7%)	8 (10.0%)	
15. Satisfaction with patient pool	Very Dissatisfied	1 (2.0%)	2 (3.5%)	3 (4.6%)	2 (2.7%)	0 (0.0%)	2 (2.3%)	2 (13.3%)	2 (2.5%)	<0.01
	Dissatisfied	3 (5.9%)	12 (20.7%)	19 (28.8%)	12 (16.4%)	4 (5.6%)	12 (13.6%)	2 (13.3%)	11 (13.8%)	
	Neutral	27 (52.9%)	30 (51.7%)	32 (48.5%)	43 (58.9%)	40 (56.3%)	52 (59.1%)	8 (53.3%)	35 (43.8%)	
	Satisfied	18 (35.3%)	12 (20.7%)	11 (16.7%)	13 (17.8%)	26 (36.6%)	21 (23.9%)	4 (26.7%)	26 (32.5%)	
	Very Satisfied	2 (3.9%)	2 (3.5%)	1 (1.5%)	3 (4.1%)	1 (1.4%)	1 (1.1%)	1 (6.7%)	6 (7.5%)	

Table 5 compares the professional attitudes of UASLP and BUAP students regarding their comfort with patients with special needs. A statistically significant difference was found in Item 16, "I feel comfortable treating patients with special needs" (p = 0.000006), with BUAP students reporting higher levels of comfort and confidence compared to their UASLP counterparts, who more often selected neutral or disagree responses. However, no significant difference was observed in Item 17, "I feel comfortable having patients with special needs as part of my patient population" (p = 0.053), indicating that students from both institutions share similar levels of acceptance toward including patients with special needs in their future practice, even if their perceived readiness to treat them differs.

**Table 5:** Professional attitudes “N” indicates the number of students in each academic year group. Percentages (%) are calculated using the total responses per academic year as the denominator. Values are expressed as frequency and percentage: N (%). UASLP: Universidad Autónoma de San Luis Potosí; BUAP: Benemérita Universidad Autónoma de Puebla

Category	Subcategory	UASLP – 3rd year (N = 51)	UASLP – 4th year (N = 58)	UASLP – 5th year (N = 66)	UASLP – 6th year (N = 73)	BUAP – 3rd year (N = 71)	BUAP – 4th year (N = 88)	BUAP – 5th year (N = 15)	BUAP – 6th year (N = 80)	p-value
16. I feel comfortable treating patients with special needs	Strongly Disagree	2 (3.9%)	1 (1.7%)	2 (3.0%)	2 (2.7%)	0 (0.0%)	1 (1.1%)	1 (6.7%)	3 (3.8%)	<0.01
	Disagree	7 (13.7%)	8 (13.8%)	13 (19.7%)	15 (20.6%)	1 (1.4%)	2 (2.3%)	0 (0.0%)	8 (10.0%)	
	Neither Agree nor Disagree	20 (39.2%)	28 (48.3%)	24 (36.4%)	29 (39.7%)	27 (38.0%)	38 (43.2%)	8 (53.3%)	21 (26.3%)	
	Agree	18 (35.3%)	15 (25.9%)	19 (28.8%)	22 (30.1%)	38 (53.5%)	39 (44.3%)	3 (20.0%)	38 (47.5%)	
	Strongly Agree	4 (7.8%)	6 (10.3%)	8 (12.1%)	5 (6.9%)	5 (7.0%)	8 (9.1%)	3 (20.0%)	10 (12.5%)	
17. I feel comfortable having patients with special needs as part of my patient population	Strongly Disagree	1 (2.0%)	2 (3.4%)	3 (4.6%)	2 (2.7%)	0 (0.0%)	4 (4.6%)	0 (0.0%)	3 (3.8%)	>0.05
	Disagree	5 (9.8%)	5 (8.6%)	11 (16.7%)	17 (23.3%)	5 (7.0%)	7 (8.0%)	1 (6.7%)	7 (8.8%)	
	Neither Agree nor Disagree	17 (33.3%)	27 (46.6%)	21 (31.8%)	26 (35.6%)	27 (38.0%)	37 (42.0%)	7 (46.7%)	21 (26.3%)	
	Agree	23 (45.1%)	15 (25.9%)	19 (28.8%)	22 (30.1%)	28 (39.4%)	35 (39.8%)	4 (26.7%)	38 (47.5%)	
	Strongly Agree	5 (9.8%)	9 (15.5%)	12 (18.2%)	6 (8.2%)	11 (15.5%)	5 (5.7%)	3 (20.0%)	11 (13.8%)	

The chi-square analysis for Table 6 revealed a statistically significant difference in behavioral intentions between UASLP and BUAP students regarding the inclusion of patients with special needs in their future professional practice (p = 0.00095). This suggests that BUAP students are more likely to express stronger intentions to include or treat patients with special needs in their careers compared to students from UASLP.

**Table 6:** Behavioral intentions “N” refers to the number of students per academic year. Percentages (%) represent the proportion of responses within each academic year. UASLP: Universidad Autónoma de San Luis Potosí; BUAP: Benemérita Universidad Autónoma de Puebla

Category	Subcategory	UASLP – 3rd year (N = 51)	UASLP – 4th year (N = 58)	UASLP – 5th year (N = 66)	UASLP – 6th year (N = 73)	BUAP – 3rd year (N = 71)	BUAP – 4th year (N = 88)	BUAP – 5th year (N = 15)	BUAP – 6th year (N = 80)	p-value
18. I will include/treat special needs patients in my future practice/professional life	Strongly Disagree	0 (0.0%)	0 (0.0%)	1 (1.5%)	0 (0.0%)	1 (1.4%)	1 (1.1%)	0 (0.0%)	0 (0.0%)	<0.01
	Disagree	5 (9.8%)	6 (10.3%)	7 (10.6%)	15 (20.6%)	3 (4.2%)	7 (8.0%)	1 (6.7%)	1 (1.3%)	
	Neither Agree nor Disagree	9 (17.7%)	20 (34.5%)	16 (24.2%)	27 (37.0%)	10 (14.1%)	22 (25.0%)	6 (40.0%)	15 (18.8%)	
	Agree	28 (54.9%)	20 (34.5%)	26 (39.4%)	21 (28.8%)	40 (56.3%)	36 (40.9%)	4 (26.7%)	34 (42.5%)	
	Strongly Agree	9 (17.7%)	12 (20.7%)	16 (24.2%)	10 (13.7%)	17 (23.9%)	22 (25.0%)	4 (26.7%)	25 (31.3%)	

## Discussion

The inclusion of people with disabilities in oral healthcare is both a public health imperative and a professional responsibility for dental practitioners. As demonstrated by the present study, significant gaps persist in dental students’ perceptions and preparedness regarding their ability to provide competent and inclusive care to this underserved population. While global discourse on health equity continues to evolve, institutional training and professional attitudes toward patients with disabilities must advance concurrently to ensure that future dental professionals are equipped to address these critical needs.

The findings of our study partially align with those of Gupta et al. (2021), who assessed the knowledge and attitudes of Indian dental students toward patients with SHCN. Gupta and colleagues reported generally positive attitudes and a moderate level of knowledge, with 60% of respondents affirming that their curriculum addressed special care dentistry (SCD); however, only 35% reported receiving practical instruction in managing such patients. In contrast, our study examined not only attitudes but also perceived preparedness, satisfaction with training, and future behavioral intentions, thereby offering a broader perspective on educational experiences. While Gupta et al. found that Indian students recognized the importance of treating patients with SHCN, our data revealed notable institutional differences between Mexican dental schools (UASLP and BUAP), particularly regarding clinical confidence and satisfaction with clinical experience. These findings suggest that, although attitudes may be generally favorable across international contexts, curriculum structure and clinical exposure remain critical factors influencing student readiness to deliver inclusive care [14].

The findings of Ramadhani et al. (2024) from Universitas Indonesia revealed that although the majority of students were aware of SCD, only a small proportion (6.1%) had received clinical training, and just 31.6% felt confident working independently with special-needs patients after graduation. Similar to the Indonesian study, we identified both didactic and clinical deficiencies in disability-related training, accompanied by a shared recommendation to strengthen undergraduate curricula and provide structured postgraduate opportunities in SCD to ensure that future dental professionals are adequately prepared to deliver inclusive care [15].

The findings of our study are also consistent with those reported by Asiri et al. (2024) in Saudi Arabia, particularly concerning the perceived lack of preparedness among dental students to treat patients with disabilities. In both studies, over 40% of students reported feeling inadequately prepared, and a strong majority emphasized the need for additional education in this area-88.5% in the Saudi study, with a similar trend observed in our Mexican sample. While Asiri et al. reported a preference for clinical over theoretical training (70.1%), our findings suggest that accommodating this preference may be challenging within the current Mexican curriculum, which remains predominantly didactic [16].

In contrast to the findings of Silva et al. (2024), who assessed Brazilian dental students’ knowledge of autism spectrum disorder (ASD) and reported generally satisfactory scores across all semesters, our study adopted a broader focus on perceptions, attitudes, and behavioral intentions toward treating patients with disabilities. While Silva et al. found no significant differences across academic years, our results revealed clear institutional differences between UASLP and BUAP in perceived preparedness, satisfaction, and comfort levels - all statistically significant. Moreover, although Silva et al. identified prior exposure to ASD as a key determinant, our data suggest that curriculum structure and overall educational experience may exert a stronger influence on students’ readiness to deliver inclusive care [17].

The study by Gómez-Vilcapoma et al. (2024), along with our research, highlights a generally poor perception among dental students regarding the management of patients with disabilities. In their study, over 85% of both interns and professors demonstrated poor perception, with a significant gender difference-female students were 41% more likely to exhibit poor perception (p = 0.028) - a pattern not observed in our findings. Unlike Gómez-Vilcapoma et al., who reported no significant influence of prior experience with individuals with disabilities, our results suggest that increased clinical exposure-particularly among BUAP students-was associated with more positive attitudes and stronger behavioral intentions to treat these patients [18].

The findings of Vainio et al. (2011) closely align with those of our study in underscoring the importance of structured education and clinical exposure in shaping dental students’ confidence and professional intentions toward patients with SHCN. Both studies demonstrate that, although students widely recognize the importance of learning to treat individuals with disabilities, their satisfaction with educational preparation-particularly clinical experience-remains moderate to low. Notably, Vainio et al. found that students’ confidence and intentions to include SHCN patients in future practice correlated significantly with the perceived quality of their education and satisfaction - a trend mirrored in our findings, especially among BUAP students. Furthermore, both studies emphasize that didactic content alone is insufficient; experiential and extramural learning opportunities are essential to foster genuine preparedness and willingness to serve this population. Our study builds on these conclusions by offering a binational comparison, confirming that significant institutional differences in training approaches can directly influence student readiness and behavioral intentions [13].

An important contextual factor that may help explain the observed differences between UASLP and BUAP students is the geographic and institutional setting of each dental school. UASLP is situated in an urban academic environment with broader access to specialized health services and a diverse patient population-conditions that may paradoxically limit students’ exposure to patients with disabilities due to more fragmented care pathways. In contrast, BUAP operates in a more rural or semi-urban setting, where students are more likely to encounter underserved populations, including individuals with disabilities, through integrated, community-based clinical experiences. This difference in clinical context may contribute to the higher levels of comfort, confidence, and behavioral intention reported by BUAP students, whose training is likely shaped by more direct interaction with vulnerable populations and a deeper understanding of access barriers and care needs [19].

Several implications for dental education emerge from this study. First, there is a clear need to incorporate structured, longitudinal content on disability care into dental curricula, beginning in the early years and extending through clinical practice. This content should not be limited to isolated lectures or passive learning but should include case-based discussions, clinical rotations involving patients with disabilities, and opportunities for reflective learning [20]. Second, faculty development is essential; instructors must be equipped with both the pedagogical tools and clinical competence necessary to mentor students effectively in SCD. Third, student feedback and perception data should be routinely collected and analyzed to support continuous improvement in curriculum design and implementation [21].

This study also carries important implications for policy. Accreditation bodies and curriculum regulators should consider establishing minimum standards for disability-related education within dental programs [22]. In addition, partnerships between dental schools and local organizations serving individuals with disabilities can offer valuable real-world exposure while simultaneously addressing community needs [23]. Interprofessional collaboration - with medical, psychological, and rehabilitative professionals - may further enhance students’ understanding of holistic care and support the development of collaborative practice models [24].

Study limitations

This study has several limitations. First, although the questionnaire achieved a high response rate and was administered across two institutions with distinct contextual characteristics, its cross-sectional design limits the ability to establish causal relationships between educational experiences and students’ behavioral intentions [25]. Second, although the survey instrument was adapted and validated based on previous studies, the findings rely on self-reported data, which may be subject to response, particularly social desirability bias [26]. Third, although the sample included students from both urban and semi-urban institutions, the results may not be generalizable to all dental schools in Mexico or internationally, particularly those with differing curricular structures or patient demographics. Lastly, while the analysis identified significant institutional differences, it did not examine other potentially influential variables such as faculty characteristics, prior volunteer experience, or specific curricular content, all of which could offer further insights into improving disability-related education. Future research should adopt longitudinal or mixed-methods approaches to assess the development of attitudes and competencies throughout dental training [27].

## Conclusions

In conclusion, while both UASLP and BUAP demonstrate a commitment to training students in the care of patients with disabilities, significant differences exist in perceived preparedness, training satisfaction, and behavioral intentions. BUAP students consistently reported greater comfort, higher training satisfaction, and stronger intent to include patients with special needs in their future practices. These findings underscore the importance of aligning curriculum, clinical training, and professional values to equip future dental practitioners with the competencies required for inclusive, ethical, and effective care. As the healthcare landscape evolves, dental education must adapt to meet the challenges and responsibilities of providing care to all patients-especially those who have been historically marginalized or underserved.

## Appendices

**Table 7:** Final version of the questionnaire used in the study (English translation) UASLP: Universidad Autónoma de San Luis Potosí; BUAP: Benemérita Universidad Autónoma de Puebla

Section	Item No.	Question / Statement	Response Format
I. Demographic Information	1	Age	Open-ended (in years)
	2	Sex	Male / Female / Prefer not to say
	3	Academic year	3rd / 4th / 5th / 6th year
	4	University	UASLP / BUAP
II. Perceptions and Satisfaction	5	My dental school has a genuine interest/concern for treating patients with special needs.	5-point Likert scale: Strongly Disagree – Disagree – Neither Agree nor Disagree – Agree – Strongly Agree
	6	My classes prepared me well to treat patients with special needs.	5-point Likert scale
	7	The school’s clinics provide a sensitive environment for treating patients with special needs.	5-point Likert scale
III. Education-Related Attitudes	8	The curriculum should include more education about treating patients with special needs.	5-point Likert scale
	9	It is very important to educate students about the treatment of patients with special needs.	5-point Likert scale
	10	I would like to have one more year as a resident before I feel comfortable treating patients with special needs.	5-point Likert scale
IV Satisfaction with various aspects of education about treating patients with special health care needs	11	Satisfaction with classroom experience.	5-point Likert scale
	12	Satisfaction with clinical experience.	5-point Likert scale
	13	Satisfaction with extramural experience.	5-point Likert scale
	14	Satisfaction with faculty expertise.	5-point Likert scale
	15	Satisfaction with patient pool.	5-point Likert scale
V. Professional attitudes	16	I feel comfortable treating patients with special needs.	5-point Likert scale
	17	I feel comfortable having patients with special needs as part of my patient population.	5-point Likert scale
VI. Behavioral intentions	18	I will include/treat special needs patients in my future practice/professional life.	5-point Likert scale
